# Death of Dr. Moses Bristol—Action of the Erie County Medical Society

**Published:** 1869-11

**Authors:** 


### Death of Dr. Moses Bristol—Action of the Erie County Medical Society.

[Abstract of the Report of the Secretary, Dr. M. G. POTTER.]

The Vice-President, Dr. MINER, announced the death of Dr. MOSES BRISTOL, and, after speaking briefly of his life and character, called for such action as the Society might please to take.

Dr. WINNE spoke of him feelingly and appropriately, referring to his useful life and hopeful death.

Dr. PRATT said that Dr. BRISTOL was born in Clinton, Oneida Co., N. Y., Oct. 21st, 1790; entered the Sophomore Class of Yale College in 1810, and graduated 1813. He subsequently attended medical lectures at New Haven, and in 1817 commenced the practice of medicine with Dr. HASTINGS in his native place. In 1822 he removed to this city, where he continued the practice of his profession until 1849, when, on account of failing health, he was compelled to relinquish it. He was a man of many virtues and few vices. Mild in disposition, he lived greatly in retirement, and was consequently little known to our younger brethren and younger citizens. His practice was not extensive, but quite respectable. His health, for the past few years, has not been good—he has recently had two attacks of paraplegia. Yesterday morning he took his breakfast as usual, and was found soon after in a paraplegic condition, which terminated his life in about eighteen hours. A fear of the pains of death has been many times expressed by our departed brother; and it seems a just reward to his excellent character, that he was allowed to pass away without a struggle or pang. He retained his intelligence remarkably with advancing years. Dr. PRATT then moved that a committee of three be appointed to draft resolutions suitable to the occasion Drs. PRATT, HARVEY and WINNE, were constituted that commttee, who presented the following:

*Whereas,*—It has pleased Divine Providence to remove from our midst our brother, Dr. MOSES BRISTOL, who, for half a century, has borne the character of an honest, prudent and faithful practitioner of medicine; and who has done much, by precept and example, to give an elevated character to the profession of our city; and who has endeared himself, to a large circle of friends, as a man of irreproachable action and principle; therefore,

*Resolved,* That the sympathies of the members of this society are tendered to his bereaved family.

*Resolved.* That these resolutions be entered upon the records of this society; that a copy be presented to the family of the deceased, and to the medical and secular journals of this city for publication.

Drs. TROWBRIDGE, HARVEY, WYCKOFF and MIXER spoke of his high character and great worth as a citizen and physician, and of his efforts to harmonize and elevate the profession.

———:o:———

Our thanks are due to the editors of the *Buffalo Evening Post* for the regular receipt of their attractive and welcome sheet.

